# Improved and Sustained Graduate Programs Diversity Outcomes: a 10-year Analysis and Summary of the Brown University IMSD Program

**DOI:** 10.1007/s41979-021-00057-z

**Published:** 2021-07-08

**Authors:** Andrew G. Campbell, Nancy L. Thompson, Marlina Duncan, Elizabeth O. Harrington

**Affiliations:** 1 Offices of the Provost and the Dean of the Graduate School, Brown University, Providence, RI 02912, USA; 2 Department of Molecular Microbiology & Immunology, Brown University, Providence, RI 02912, USA; 3 Brown IMSD Program, Division of Biology & Medicine, Brown University, Providence, RI 02912, USA; 4 Department of Medicine, Brown University, Providence, RI 02912, USA; 5 Research Division, Providence VA Medical Center, Providence, RI 02908, USA

**Keywords:** Graduate student training, Education, Diversity, Underrepresented minority

## Abstract

This report describes the 10-year outcome of implementing practices that support and foster success of underrepresented students in science, technology, engineering, and math (STEM) graduate training at Brown University. The results show sustained improvements in compositional diversity, retention, and degree attainment of supported students relative to their peers. Among the outcomes is an increase in enrolled student diversity from 19 (35 of 179) to 26% (58 of 223) for historically underrepresented minority (URM) students and an increase in Ph.D. degree attainment from 4 (1 of 25) to 14% (6 of 44) for this group. These achievements follow the introduction and coordination of academic and co-curricular practices through the National Institutes of General Medical Sciences–funded Brown University Initiative to Maximize Student Development (IMSD) Program. At the center of these outcomes is the alignment of IMSD practices with recent diversity initiatives launched by the university. The outcomes described result from long-term commitments to building a culture that includes: (1) development of relationships that serve underrepresented students, (2) provision of a personalized education program of support and skills-based learning that supplements discipline-based research and coursework, and (3) investments in processes that build a culture that values and benefits from diversity. These practices may yield similar outcomes and success for students when applied elsewhere.

## Introduction

Women, racial, and ethnic minorities comprise ~66.7% of the US population and ~65% of its 18–64-year-old domestic workforce (NSF Table 1-2, 2017). These groups however account for only ~47% of the Science, Technology, Engineering, and Mathematics (STEM) employed workforce (NSF Table 9-7, 2017) and thus make up the underutilized and underrepresented US majority. Women alone comprise 51% of the population but continue to be underrepresented in many academic and nonacademic STEM fields. The circumstance for underrepresented racial and ethnic minorities who make up 27.9% of the US population is far worse. They account for the largest underutilized group in the US workforce on a proportional basis (National Academies, 2014; NSF-Table 9.7, 2017), accounting for 13% of employed Science & Engineering (S&E) degree holders working in STEM. In addition to the racial and ethnic basis of their underrepresentation, the groups are also often underrepresented based on their socioeconomic “disadvantaged” status.

Several programs have been developed to address poor STEM field diversity at the undergraduate and graduate levels (http://sloanphds.org/.; https://www.hhmi.org/science-education/programs.; https://meyerhoff.umbc.edu/.; https://www.nigms.nih.gov/training/IMSD). Within the field, a number of reports document practices and outcomes that show what is achievable with investments in STEM education, mentoring, and training (Hitchcock et al., 2017; Jomoke Ladeji-Osias et al., 2015; Prunuske et al., 2017; Sorkness et al., 2017; Toven-Lindsey et al., 2015; Williams et al., 2017; Williams et al., 2021). Work within the field that integrates the theory and practice of diversifying STEM fields serves as guiding principles and as a blueprint that applies to both graduate and undergraduate training (Byars-Winston et al., 2011). Undergraduate college-level programs provide underrepresented (UR) students with access to opportunities where few or no opportunities previously existed. In contrast, graduate-level programs operate primarily to strengthen student readiness for careers in STEM fields. Collectively, these programs respond to unfulfilled expectations of student readiness by equipping them with skills that fill knowledge gaps in prior education and training. These programs also help to address individual student issues such as self-esteem. They also help to raise individual internal locus of control (Vansteenkiste et al., 2006) which shapes individual self-determination and plays an important role in individual success. In short, they function to negate factors that threaten student persistence and retention by harnessing aptitude, addressing stereotype threat (Steele and Aronson, 1995), and building competencies. Systematically, more attention must also be given to the broader challenges of managing infrastructure barriers that impact student persistence and success. These challenges include the pressures of rising faculty workload which increases administrative burdens at a cost to student mentoring and training (National Academies, 2012). Other challenges include the need to reimagine or abandon the outmoded conceptualization of the STEM pipeline (Allen-Ramdial and Campbell, 2014; Estrada et al., 2016) and the need to change the current ethos and practices of the academic community (Alberts et al., 2014). These challenges continue to foster pessimism and discourage students from entering STEM fields in the academy and beyond, and their effects are greatest on underrepresented students. Disproportionate underrepresentation of UR students in the graduate ranks also persists because the talent pools from which they are drawn continue to be limited. Increasing UR student representation will therefore require greater investments in minority-serving institutions (MSIs) and community colleges where disproportionately larger numbers of these students are educated (Knapp et al., 2012; NCPPHE, 2011). As these institutions increasingly become the primary source pools of the general future US workforce, they should also become the talent source pool of future advanced degree scholars.

Improved educational and training practices that minimize and erase the barriers UR students face when entering STEM fields should be supported. Such practices should recognize that not all problems of underrepresentation are problems of student abilities and motivation. Systemic institutional flaws exist as barriers to success, and they operate independently of individual capacity and desires to pursue STEM field careers. Approaches that address challenges to broadening participation that are limited to managing “the individual’ without simultaneously eliminating and managing systemic flaws will likely yield limited benefits. The challenges for higher education, therefore, are to do as good a job recognizing and erasing barriers to academic pursuit and degree attainment that exist in organizational and operational practices as are being taken to identify and address gaps in student readiness to pursue their chosen careers.

In 2008, Brown University established the Initiative to Maximize Student Development (Brown IMSD, n.d.) program to increase the number of UR students, particularly racial and ethnic minorities among the doctoral programs in the Division of Biology and Medicine (BioMed) (Thompson and Campbell, 2013). Underrepresented Minority (URM) is inclusive of individuals as defined by the National Science Foundation (National Academies, 2014) who are underrepresented by their race and ethnicity in the US population and are simultaneously US citizens or permanent residents. This group includes Hispanics/Latinos, American Indians or Alaska Natives, Black/African Americans, and Native Hawaiian or Pacific Islanders. At Brown, individuals who are of two or more races or ethnicities of which one race or ethnicity is a URM are also classified as a URM. The broader term “underrepresented group” or UR” group collectively refers to URMs as well as other non-majority racial and ethnic groups. The UR group classification also includes women, Asians, individuals of nonbinary gender identities, and other circumstantially disadvantaged individuals such as those with disabilities whose representation in STEM is lower than their representation in the US population.

Brown IMSD’s work is directed toward increasing graduate-level diversity, and this objective aligns with the university’s mission to serve the community and nation in preparing students to “discharge the offices of life with usefulness and reputation” (https://www.brown.edu/about/brown-glance ). This objective further aligns with the mission of the National Institute of General Medical Science’s IMSD initiative (https://www.nigms.nih.gov/training/IMSD) to diversify the STEM workforce.

The aims of this work were (1) to assess recent gains in graduate student compositional diversity associated with IMSD program support and Brown University diversity interventions; (2) to compare IMSD student retention, degree completion, and achievements relative to non-IMSD peers; and (3) to provide a dataset to aid in planning and administering future diversity measures in STEM graduate training at Brown University.

This paper describes Brown IMSD’s work over 10 years, and outcomes serve as a basis for expanding the program from serving 9 STEM Ph.D. programs in BioMed to serving all STEM Ph.D. programs across the university.

## Methods

### Participants

#### Selection of IMSD and Control Groups

IMSD trainees are Ph.D. students who receive financial support from the program for a minimum period of 1 year. This financial support is typically provided to students at the early graduate career stage. IMSD trainees transitioning to other financial support after 1 year continue to be identified as IMSD program-affiliated trainees until completion of their Ph.D. degrees. All IMSD trainees are UR students who have been determined by their graduate programs to benefit from participation in IMSD program activities. From 2008 to 2018, 66 students have been supported by IMSD with 28 receiving the Ph.D. degree and 3 earning the master’s degree. From 2008 to 2018, matriculating and matriculated IMSD trainees were matched to 122 non-IMSD trainees by year of entry and Ph.D. program of entry at Brown. This matched group of trainees is referred to as non-IMSD matched cohort. Each IMSD trainee is matched to at least 1 non-IMSD trainee but no more than 9 non-IMSD trainees. In 2008, in one Ph.D. program, Molecular Pharmacology and Physiology, the IMSD trainee to matched cohort ratio was 1: 1 ratio, with an entering class of 2 IMSD trainees and 2 non-IMSD trainees. In all other years, each matriculating IMSD trainee was matched to >1 but no more than 9 non-IMSD trainees. This match variability is the result of differences in the matriculating class size of each graduate program. From 2008 to 2018, the progress of 394 BioMed non-IMSD, 1715 university STEM, and 3179 university active or completed Ph.D. students was also monitored. All Brown University Ph.D. students receive the same base level of financial support including stipend, tuition, and health benefits. Accordingly, economic status is not a pre-qualifying criterion for IMSD trainee status.

#### Participating Ph.D. Programs

**Fig. 1:**
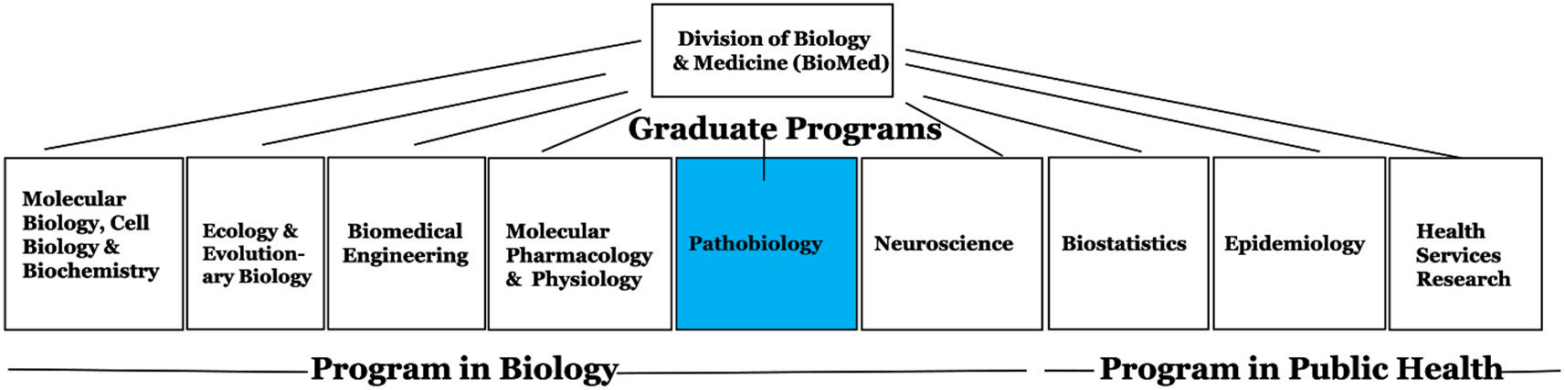
Founding IMSD partnering Ph.D. programs. Listing of the original 2008 IMSD partner Ph.D. programs in the Division of Biology and Medicine (BioMed). The public health programs are currently housed outside of BioMed in the School of Public Health. The original program in which IMSD began, Pathobiology, is shown in the shaded blue box

The Ph.D. programs participating in IMSD from 2008 to 2018 included all 9 programs in the Division of Biology and Medicine (BioMed) at Brown. The BioMed division is comprised of the Warren Alpert Medical School and 5 campus-based basic sciences departments and their affiliated graduate programs. Ph.D. training faculty include faculty in the Alpert Medical School and Brown University affiliated hospitals and the basic sciences departments. Prior to 2013, the BioMed division was composed of the Programs in Biology and Programs in Public Health which included the Ph.D. programs as shown in Figure 1. These programs represent the founder IMSD participating Ph.D. programs. In 2013, the Programs in Public Health became the independent School of Public Health (SPH). The 10-year BioMed IMSD data presented here includes data for the original public health Ph.D. programs that now reside in the School of Public Health.

### Procedures

#### IMSD Programming and Activities

Since 2008, IMSD has put in place several practices designed to maximize student success in STEM field Ph.D. programs at Brown (Thompson and Campbell, 2013). These practices support graduate student academic preparation and achievements, addressed social climate issues, and built a sense of belonging. The major co-curricular practices of the program including the use of skills-based training modules, advising and research progress assessment, partnership development, and student community meetings have been previously described (Thompson and Campbell (2013) and Supplemental Figure 1 (Online Resource 1)).

#### Institution-Wide Diversity Programming

Expansion and creation of new institution-wide diversity programming in 2016 coincided with the transition of the BioMed IMSD program to the institution-wide IMSD program. A detailed description of programming and the frequency of their offerings are summarized in Supplemental Figure 2 (Online Resource 2).

#### Coordination of IMSD, Diversity and Inclusion Action Plan (DIAP), and Graduate School Programming

The transition of the BioMed IMSD program to an Institutional IMSD Program entailed expanding the program to 14 additional STEM Ph.D. programs engaged in biologically and biomedically related studies across the university. This expansion aligns with the goal of preparing a workforce that draws on and benefits from changing US demographics to advance human health and national scientific productivity. At Brown University, graduate training in STEM is highly interdisciplinary and supported by strong cross-connections between our individual schools, departments, and programs. This interdisciplinarity means that all 23 participating IMSD Ph.D. programs engage in biologically, biomedically, and/or public health–relevant research training and education. Participating faculty include those in the Division of BioMed and in the Schools of Engineering and Public Health. A sizeable number of faculty in these schools and programs also serve as trainers on other institutional training grants that are often housed outside of their home programs, departments, and schools. Expansion of the IMSD program was collaboratively overseen by the IMSD PIs, the Graduate School, as well as the Office for Institutional Equity and Diversity.

#### “Preview Day”: Network-Building Opportunities for Prospective Graduate Students

IMSD Partner’s Day was launched in 2008 as the forerunner of Preview Day. Preview Day, which has been piloted since 2017, has contributed to increases in the numbers of URM student applicants and matriculants. In 2017, 55 prospective URM graduate students from predominantly minority-serving institutions were hosted at Brown to expose them to the graduate culture and climate across our graduate programs. Of this number, 21 (38%) subsequently applied for graduate studies, and 19% were admitted as part of the entering 2018 class of Ph.D. students. In 2018, 28 of 125 (22.4%) STEM students applying to attend Preview Day were selected to attend. Twenty-seven of these subsequently applied for graduate studies, 9 were admitted, and 7 eventually matriculated, accounting for 12% of all matriculating URM Ph.D. students as part of the entering class of 2019–2020. Over the past few years, Preview Day has yielded more applicants to our graduate programs than any other outreach efforts, including engagement of prospective graduate students at scientific conferences.

#### “Super Monday” Visits to Brown

Super Monday visits have played an important role in admitted URM Ph.D. students ultimately selecting Brown University as their first choice for graduate studies. Participation in the event by admitted students serves as a strong predictor of eventual matriculation at Brown. Over the 2 most recent admissions cycles, for example, an average of (27) 87% of admitted URM students electing to attend Super Monday enrolled into Brown Ph.D. programs to which they were accepted. These acceptances typically follow student visits to Brown, and the majority of attendees report that their Super Monday experience helped them to make their final decision to attend Brown. This outcome contrasts with students who were offered graduate admissions but did not participate in Super Monday. Of these, only 37% elected to enroll in graduate studies at Brown.

#### Data Collection and Analysis

Ph.D. student enrollment and retention data, as well as student publications and awards, were analyzed for the period 2008–2018, corresponding to the first 10 years of IMSD program operations. URM student applications, as well as admissions and matriculation data for the university including BioMed and SPH, were analyzed over 6 years (2014–2019). The 2014–2016 period corresponds to the 3 years preceding the 2016–2017 spring semester conversion of BioMed IMSD to the institution-wide IMSD program. This transitional period coincided with the launch of Brown University’s Diversity and Inclusion Action Plan (DIAP, n.d.) and new Graduate School diversity programming. Ph.D. student post-training placements were tracked using various methods, including LinkedIn and alum self-reported data. Time to the Ph.D. degree at Brown is calculated as the time from first program enrollment to the time of completion of Ph.D. degree requirements, including successful defense of the Ph.D. thesis. All data reported for 2008–2018 in this study use the next earliest May graduation date as the time of degree completion. Attrition is defined as departure from Ph.D. training before completion of the Ph.D. or master’s degree. Nonparametric analyses of all data were completed to evaluate statistical significance of group differences. Unpaired t-test analyses of GRE scores, GPA, and publications were completed using GraphPad Prism analysis software. Analysis of variance (ANOVA test) was performed to determine the significance of the difference in time to the Ph.D. degree and publications between the IMSD cohort, matched BioMed non-IMSD cohort, and the BioMed non-IMSD cohort. ANOVA testing was also done to determine the significance of the difference in Ph.D. attrition rates.

Publication searches were performed on the National Library of Medicine’s MEDLINE publicly available database using the PubMed interface at https://www.ncbi.nlm.nih.gov/pubmed?myncbishare=brownu. The search string used a Boolean combination of each student’s last name, crossed with “Brown University” to return the maximum number of results, and was limited to the years 2008–2018. Searches for publications by the 31 IMSD trainees yielded 767 citations and 3228 citations for the 122 matched group. The search results were subsequently screened and filtered for citations with a combination of all of the following criteria: correct author first name or first initials, Brown University faculty affiliation in one of the programs listed in Figure 1 at the time of publication, graduate program affiliation, and relevant biomedical, life science, and public health research topics. Students publishing collaborative manuscripts in their field of studies independent of their faculty advisors were also retained. Because publications did not always cite grant support, this criterion was eliminated from the search parameters.

Data on federal graduate fellowships awarded from 2008 to 2018 was retrieved by searching the publicly available NIH and NSF databases using the Research Portfolio Online Reporting Tools (RePORT) at https://report.nih.gov/ for NIH database searches and the NSF database search tool at https://www.nsf.gov/awardsearch/. Results were confirmed by cross-checking institutional records.

Data on Ph.D. program attrition and retention rates, time to degree, GPAs, and GRE scores were gathered from institutional records and de-identified for analysis.

In addition to documenting formative and summative measures of student academic success, climate surveys are administered to enable IMSD to gauge student perception about the program’s impact on institutional diversity climate. IMSD surveys are administered to all Ph.D. students across the university. These are administered annually as anonymous web-based surveys by external evaluators, Meridian Solutions Inc., and DePass Consulting.

## Results

Early analysis of our IMSD program was completed in 2013 and confirmed the benefits of the program (Thompson and Campbell, 2013). Having described the program’s initial implementation and success, the current analysis examines the longer-term benefits and new initiatives over an extended 10-year period from 2008 to 2018. While not designed as strictly a research study, our extensive data collection and follow-up has allowed us to provide significant statistics and correlations relative to diversity advances at Brown University.

### IMSD-Supported Growth of Enrolled Underrepresented Ph.D. Students

**Fig. 2:**
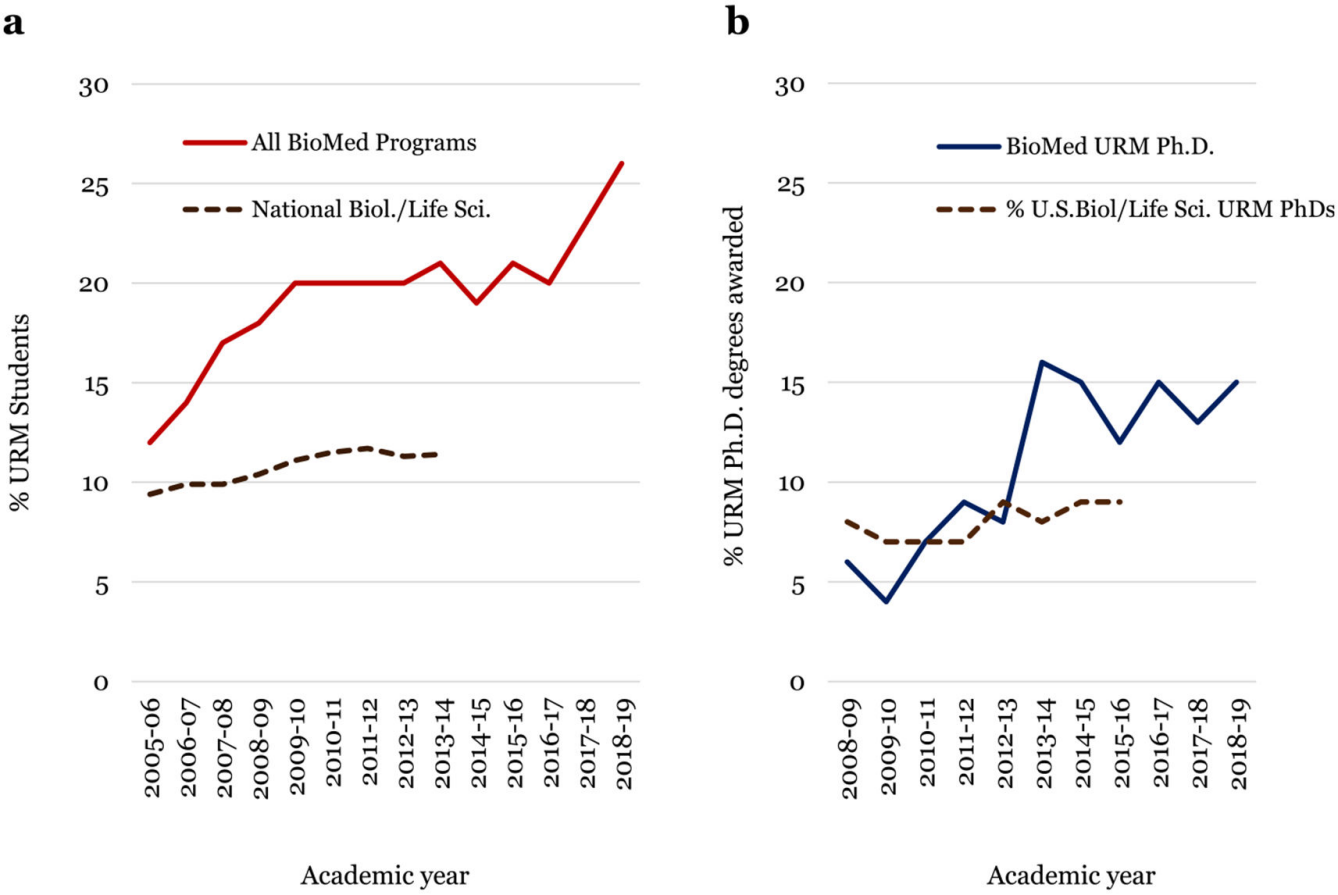
Change in URM student enrollment in Ph.D. programs and in Ph.D. degree attainment. **a** Change in percent URM students in Ph.D. programs for the period 2005–2006 to 2018–2019. Changes in URM Ph.D. student populations across the entire Division of Biology and Medicine (BioMed) and nationally are shown. National Biology/Life Science corresponds to all URM students who are US racial and ethnic minority students in Ph.D. programs in nonagricultural biological sciences disciplines (NSF Table 3-1, 2016). Percent values exclude the small numbers of students who elect not to identify their racial and ethnic backgrounds. Publicly accessible NSF data is available only through 2016. **b** Ten-year analysis of Ph.D. completion. The 10-year change in completion of Ph.D. degrees by URM trainees in Brown’s BioMed Ph.D. programs is shown. Comparisons are made to the change in national production of Ph.D. in the biological and life sciences. National Biology/Life Science corresponds to all URM students who are US racial and ethnic minority students in Ph.D. programs in nonagricultural biological sciences disciplines (NSF Table 3-1, 2016). Percent values exclude the small numbers of students who elect not to identify their racial and ethnic backgrounds. Publicly accessible NSF data is available only through 2016

The Brown IMSD program was formally established in the Division of Biology and Medicine (BioMed) at Brown in the spring semester of the 2007–2008 academic year. It was based on the success of diversity practices applied in the Pathobiology graduate program which coincided with increases in URM Ph.D. student numbers in the program (Thompson and Campbell, 2013). In 2008, these practices were expanded to 8 other Ph.D. programs in BioMed (Figure 1). By the 2008–2009 academic year, URM student representation rose to 35 of 187 (19%) from a 2005 to 2006 low of 19 of 158 (12%) (Figure 2a). This increase compares favorably to national URM graduate enrollment of ~10–12% over the same period (NSF Table 3-1, 2016). The increase in BioMed’s graduate diversity has been sustained for the past 10 years, rising to 26% (58 of 223) in 2018–2019. The most recent increase from 42 of 213 (20%) to 26% (58 of 223) for the period 2016–2018 benefitted from Brown’s expanded institutional commitment to diversity and inclusion as detailed below. Moreover, with the 2019–2020 academic year entering graduate class, BioMed URM diversity is now ~29% (data not shown), a value that aligns with URM representation in the US population (NSF Table 1-2, 2017).

### Alignment of Recruitment, Matriculation, and Support Practices Across Graduate Programs

Applicants to BioMed Ph.D. programs typically identify their desired area of study early in the admissions process, and each program utilizes its own recruitment and admissions decision-making process. While this approach provides program autonomy, it lacked standard practices to identify, engage, recruit, and support UR students, particularly URM students. Though not directly involved in recruitment, IMSD has been effective in supporting graduate program recruitment and admissions practices by assisting outreach efforts and supporting representation at regional and national conferences where large numbers of UR students present their work. The range of activities that correlate with URM Ph.D. student increases is shown in Supplement Figures 1 and 2 (Online Resources 1 and 2 respectively).

### Increase in BioMed URM Ph.D. Degree Recipients

The proportion of Ph.D. degrees awarded to URM students in BioMed in the IMSD era increased from ~4 in 2008 to 14% (6 of 44) in 2015 (Figure 2b). Between 2012 and 2015, new Ph.D. programs were established in the Program in Biology and Public Health, and the absence of diversity in these programs is reflected in the lower output of URM Ph.D. recipients from 2016 to 2018. The overall increase over the past 6–8 years however has moved Brown above the annual national average output of 8% of Ph.D.s in the life and biological science awarded to URMs over the same time period (NSF Table 7-4, 2017). The majority of Ph.D. degrees awarded in BioMed during this period were to African American trainees. Though the absolute number of degrees awarded is low, the output ranks Brown among the top 25 Carnegie-classified non-MSIs awarding Ph.D.s to African Americans in the biological and biomedical sciences (Diverse Issues in Higher Education, n.d.). This improved ranking is consistent with our reported increase in matriculation and retention of URM trainees in BioMed Ph.D. programs. All IMSD program alums are employed in fields using their degrees in the area of their training (data not shown).

### Comparison of Entrance Credentials and Attainment of Training Milestones

**Fig. 3:**
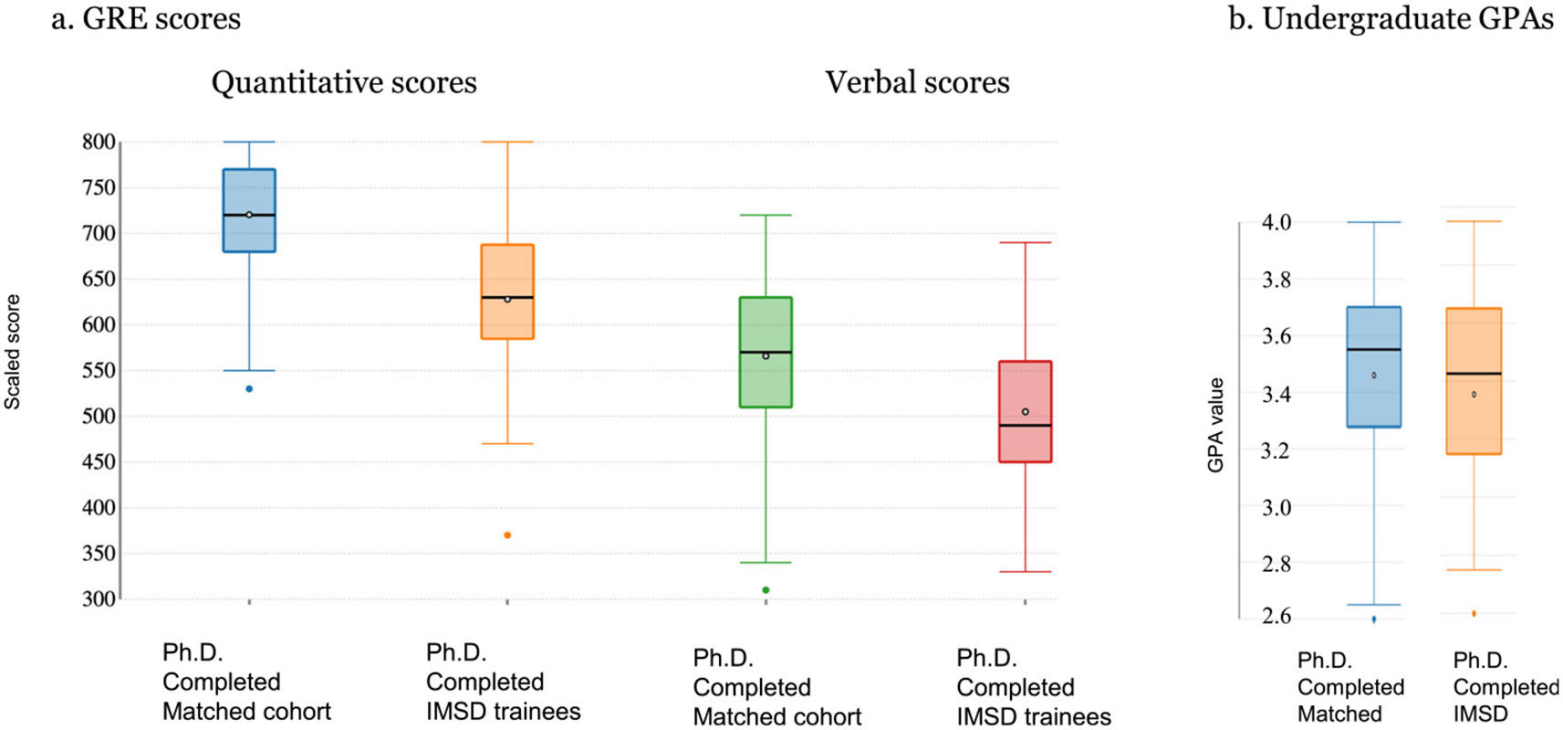
Whisker and boxplot analysis of admissions metrics of completed trainees. Data is presented for IMSD and non-IMSD matched cohort trainees who completed the Ph.D. degree between 2008 and 2018. Matched cohorts are Ph.D. trainees who matriculated into Ph.D. studies into the same program of at least 1 IMSD trainee in the same year. **a** GRE scores for 96 of 122 matched cohort compared to 31 IMSD trainees at the time of entry into Ph.D. studies. GRE scores were waived for 26 of the 122 matched cohorts who submitted MCAT scores or other equivalent standardized test scores. GRE scores were waived for Brown undergraduate applicants to the Graduate School. Among the non-IMSD matched cohort scores were scores of 8 trainees who attrited after matriculation but before completing the Ph.D. degree. IMSD trainee GRE scores include scores for 3 trainees who attrited before completing the Ph.D. degree. All GRE scores were converted to the 200–800 scoring scale used before August 2011. **b** Undergraduate GPAs were measured on a scale of 1.0–4.0, with 4.0 corresponding to an “A” and 1 to a “D.” GPAs for trainees from undergraduate institutions where no GPA value is given had GPA values computed by converting letter and mathematical grades to GPA values on the same 1.0–4.0 scale. A grades were assigned for scores of 90% and above, B for scores of 80–89%, C for scores of 70–79%, and D or failing grades for scores of 60–69%. Individual dots positioned outside of whiskers and boxes identify single outliers. The matched cohort represents the same matched group as the matched cohort of BioMed non-IMSD trainees shown in Figure 5

Figure 3 compares the entering GRE and GPA scores of a matched cohort of 122 BioMed non-IMSD Ph.D. students and 31 IMSD Ph.D. students who began and ended their graduate careers between 2008 and 2018. GRE and GPA scores have been used in making admissions decisions and as tools to predict future student success. Analysis of these scores and values show a statistically significant difference between GRE scores for IMSD trainees and the non-IMSD matched cohort. Although there is a mathematical variance in the mean and median GPA values between the two groups, the differences are not statistically significant.

### IMSD Student Retention and Degree Completion

**Fig. 4:**
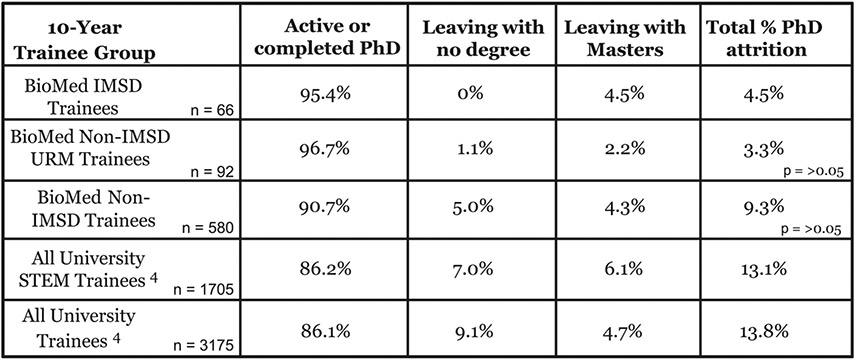
Attrition analysis of current and former PhD trainees, 2008-18 10-year analysis of Ph.D. trainee attrition and completion. The progress of IMSD trainees was compared to the progress of BioMed non-IMSD URM trainees, BioMed non-IMSD trainees, all STEM Ph.D. trainees across the university, and all Ph.D. trainees across the university. Students attriting from Ph.D. programs include those who withdrew, failed to meet academic standards, and/or left with a master’s degree before completion of the Ph.D. degree. n, Number of trainees in each of the named 10-year trainee group. ^4^Includes IMSD trainees. IMSD trainees represent students supported across the Ph.D. programs listed in Figure 1. Values are given for students enrolled between 2008 and 2018. Non-URM students include both domestic and international students. Students on leave are listed as non-attriting students. p Values given are relative to IMSD trainees

Co-curricular IMSD programming forms a supportive scaffold around trainees throughout their degree training. This programming compliments the academic training students receive in their individual graduate programs by helping to raise student readiness for academic success. Figure 4 summarizes the 10-year analysis of retention and attrition rates of current and past IMSD trainees compared to BioMed non-IMSD URMs, all BioMed non-URM Ph.D. trainees, as well as all STEM Ph.D. trainees and all Ph.D. trainees at Brown between 2008 and 2018. The results show that over this time period, the majority of IMSD and non-IMSD URM Ph.D. trainees are retained in Ph.D. studies. Although some IMSD trainees attrit before earning their Ph.D.s, all who attrit earned their master’s degrees before attriting. In contrast to this, attrition across all Biomed Ph.D. programs was calculated to be higher at 9.3% (54 of 580 students), and less than 50% of attriting students earned their master’s degree before early departure. More broadly, attrition of trainees across all STEM Ph.D. fields and from all Ph.D. programs at Brown over the 10-year period was higher at 13.1% (223 of 1705) and 13.8% (438 of 3175), respectively.

**Fig. 5:**
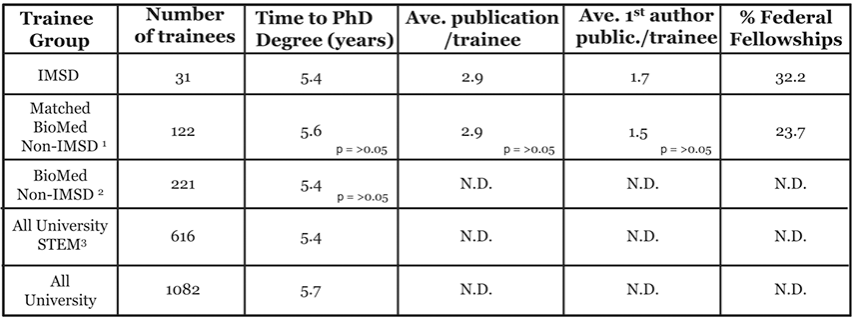
Achievements of trainees completing Ph.D training Achievements of Ph.D. trainees between 2008 and 2018. IMSD, IMSD trainees in BioMed and the School of Public Health (SPH) who have earned graduate degrees; matched non-IMSD, non-IMSD Ph.D. trainees in BioMed and SPH; non-IMSD, US and international Ph.D. trainees; all STEM trainees, Ph.D. trainees in all STEM fields, including Mathematics and Geological Sciences across the university; and all university. ^1^ Includes BioMed and SPH non-IMSD domestic and international Ph.D. graduates matched by program and year of program entry, ^2^ includes BioMed and SPH non-IMSD URMs and non-URM and international Ph.D. students and graduates across all BioMed and SPH Ph.D. programs, and^3^ includes all STEM Ph.D. trainees, except Mathematics and Geological Sciences, across the university. Twenty-eight of 31 students completed the Ph.D. degree. One hundred fifteen of 122 matched non-IMSD trainees completed the Ph.D. degree between 2008 and 2018. Two hundred twenty-one non-IMSD BioMed trainees completed the Ph.D., 23 of these left without the degree. All enrolled Ph.D. trainees completed the Ph.D. degree on May 2018. Until 2018, Brown University formally awarded advanced graduate degrees only in May of each year. The calculated time to degree and graduation date is set as the next earliest May date. Annotated and indexed publications by all trainees was gathered from PubMed collecting only published works with publication dates from 2008 to 2018 inclusively. The number of publications attributable to each group represents unique publications. To avoid double or triple counting of publications, papers co-authored by two, three, or more trainees in the IMSD and matched cohort groups were excluded. The average number of first-author publications was based on all publications attributable to all groups. Federal fellowships include National Institutes of Health F31 Predoctoral fellowships and National Science Foundation Graduate Research Fellowship Program (GRFP) fellowships. Fellowship success rate among IMSD cohort: 10 of 31 trainees receiving fellowships (95% CI [0.152, 0.487]). Fellowship success rate among matched BioMed non-IMSD cohort: 29 of 122 receiving fellowships (95% CI [0.187, 0.272]). N.D., not determined

Comparisons of the achievements of IMSD trainees and the matched BioMed non-IMSD trainees between 2008 and 2018 who have completed graduate training in 2018 are summarized in Figure 5. These include a comparison of time to the Ph.D. degree, publication numbers, and individual national fellowships won. Comparisons of time to the Ph.D. degree for IMSD trainees were also made to the larger unmatched BioMed non-IMSD trainee population as well as to all university STEM Ph.D. degree recipients and all university Ph.D. degree recipients for the period 2008–2018. Although there is a small mathematical difference in time to degree between the groups, the difference is not statistically significant. In fact, standard deviation (SD) analyses for time to degree for IMSD trainee group, matched BioMed non-IMSD trainee group, and BioMed non-IMSD trainee group were 0.96, 1.15, and 1.13, respectively, with no statistically significant difference between the values. A comparison of the data between IMSD trainees and the matched cohort shows IMSD trainees complete their Ph.D. degrees in an average time of 5.4 years compared to the 5.6 years for the 115 of 122 graduating matched cohort trainees. The remaining 7 trainees of the matched cohort failed to graduate. Comparisons of the time to the Ph.D. degree for the 221 BioMed non-IMSD, 616 university STEM, and 1082 all university Ph.D. students also showed a small mathematical variance.

Scientific publications represent another key measure of graduate student achievement and success, and the total number of annotated and indexed PubMed publications produced by IMSD trainees and their matched cohort of degree completers between 2008 and 2018 is presented in Figure 5. IMSD trainees produced an average of 2.9 publications per trainee, compared to 2.9 publications by the matched BioMed non-IMSD cohort. These average numbers were calculated with a 95% CI [−1.054, 1.006] and SD of 3.15 for the IMSD cohort and an SD of 2.43 for the matched cohort. The IMSD cohort publication number range was calculated to be 0–14, with a median number of 2 publications per trainee. This compares to a publication number range of 0–11 and a median number of 3 per matched BioMed non-IMSD trainee. First-authorship on publications often indicates a significant contribution by the lead authors in the research design, analysis, and writing of the published work. Our IMSD cohort produced an average of 1.7 first-author manuscripts per trainee compared to 1.5 first-author publications produced by each trainee of the matched cohort. This difference is not statistically significant. The mean numbers of first-author publications by the groups were calculated with a 95% CI [ −0.34, 0.95]. First-author publication number range for IMSD trainees was 0–7, with a median number of 1 first-author publication. For our matched BioMed non-IMSD trainees, the number of first-author publication range was 0–9, with a median number of 1.

The awarding of national fellowships is based on promise and potential that the recipients will develop to become among the most outstanding scientists who contribute to the US biomedical and health workforce inside and outside of the academy. Although the total numbers of awarded national fellowships are low, both IMSD and the matched cohort of non-IMSD trainees win a combination of NIH F31 and NSF Graduate Research Fellowship Program at similar rates.

### IMSD Era Increases in Graduate Applicant, Admitted, and Matriculating Students and Implementation of Brown’s Diversity and Inclusion Action Plan

The concurrent expansion of the BioMed IMSD program and implementation of Brown’s DIAP led the way to an almost immediate increase in URM student applicant numbers to the Graduate School. Beginning with the 2017 admissions cycle, more URM Ph.D. students applied, were admitted, and matriculated into Brown’s Graduate School than at any other time in the Graduate School’s 133-year history. Increases include a 45% and 87% rise in applications and admitted students, respectively, for the period 2017–2019 relative to the 2014–2016 period for both STEM and non-STEM applicants and students. This observed 3-year increase in graduate student diversity across many of our programs represents new and intentional growth of the entire graduate student population and not a reduction or replacement of other populations of students. Increase in BioMed graduate student diversity was not restricted to increases in students matriculating only from partner institutions. We believe that our ability to attract and enroll larger numbers of URMs from other institutions, however, did benefit from lessons learned from the relationships we established with our partner institutions.

### IMSD Program Trainees Express Higher Satisfaction with Institutional Climate

IMSD has used annual climate surveys since 2008 to measure satisfaction among students participating in the program. Because survey questions evaluate elements of graduate training relevant to all graduate students, responses were also solicited from non-IMSD Ph.D. students across all graduate programs in the university. This approach puts into a larger and more relevant context the responses of IMSD trainees. When non-IMSD student climate survey responses are compared to IMSD students, IMSD students express higher satisfaction with their training and departments (data not shown). IMSD students did express lower satisfaction with their development as scholars when compared with other STEM Ph.D. students. This response however was inconsistent with and contradicted responses to the question of how they are being prepared for their career goals. The likely explanation for this contradiction is that respondents expressing satisfaction with preparation toward their career goals do not see their preferred training paths as necessarily leading them to become scholars. The overall results show that independent of their individual graduate programs, IMSD trainees, in general, are more satisfied with their graduate program and graduate training than non-IMSD trainees. This is also true when we compare IMSD trainee satisfaction relative to non-STEM Ph.D. trainees.

## Discussion

The goal of Brown’s IMSD program has been to increase the numbers of underrepresented students trained in STEM fields and who join the US STEM workforce. Because racial and ethnic minorities are among the least represented in STEM fields, we have emphasized increasing their numbers in these fields using programming designed to address potential gaps in background preparation and practices that increase their sense of belonging. This emphasis however does not exclude support of students from other underrepresented groups or the participation of other students in the program and its activities.

Between 2008 and 2018, 31 former IMSD trainees completed their course of study at Brown, earning either their Ph.D. or master’s degrees. The appointment of these students as IMSD trainees was based on the determination that their participation in the program would benefit their training, offsetting perceived weaknesses that would impact their performance. These trainees earned the Ph.D. degree at rates similar to non-IMSD trainees in BioMed and across the university, publish the same number of manuscripts, and garner national fellowships at similar rates. Although only 13% (4 of 31) IMSD trainees receive their undergraduate degrees from AAU institutions, compared to 40% (48 of 122) of the matched cohort, these students perform as well as their matched counterparts. The observation that IMSD trainees begin their graduate training with GRE scores lower than their non-IMSD peers suggests that these scores are not reliable predictors of future performance—an observation that is also supported by a number of studies (Petersen et al., 2018). GRE scores appear to have little value in terms of predicting student success. Rather, these scores have been useful in diagnosing student needs as graduate students.

IMSD trainees completing training between 2008 and 2018 represented admitted students who would otherwise not have been admitted without IMSD support because they were considered not as competitive as other graduate applicants. These trainees however have succeeded at levels comparable to trainees admitted without IMSD support.

No significant difference was noted between the GPA scores of IMSD trainees and their matched cohort suggesting that this was not a factor in defining applicant strength at the time of admissions. While aggregated applicant GPA data showed no statistical difference when the GPAs of the 31 former IMSD trainees are compared to that of the 122 former matched cohort, there are noted differences in the mean, median, and interquartile range GPAs between the 2 groups. Despite differences in their credentials at the time of matriculation, IMSD trainees (including those who attrit) perform as well as their non-IMSD peers.

Results presented show that the Brown IMSD program has contributed to increasing diversity in all BioMed Ph.D. programs, raising compositional diversity to a level that is now proportional to the representation of URM in the US population. The outcome of work to date suggests that there is a direct correlation between IMSD programming and student success in graduate training. By applying IMSD program practices broadly to all graduate programs, we foresee greater increases in retention and successful student post-training placement. Although a number of factors have been reported to account for attrition (Vassil and Solvak, 2012), we are unable to define the primary factors that account for the higher attrition we observe across the university.

We believe that much of IMSD’s success is related to its close coordination with individual graduate programs and collaboration with faculty and administrators. Moreover, we have found that the participation of the target student population in multiple activities in sequence such as Brown Preview Day, followed by engagement at national conferences such as Annual Biomedical Research Conference for Minority Students (ABRCMS) and Society for Advancement of Chicanos/Hispanics and Native Americans in Science (SACNAS), and then participation in Super Monday by admitted students result in the highest yield of matriculating underrepresented students. Other efforts to increase diversity where this sequencing and coordination are absent have not achieved similar levels of diversity. An increasing number of graduate programs have begun to adopt our sequencing practices which we see as the beginning of some institutionalization of practices.

Among outcomes noted in our analysis is the lower attrition rate of IMSD trainees and non-IMSD URM students relative to all other groups (Figure 4). We believe this is attributable to IMSD student adherence to IMSD programming. For example, all IMSD trainees have in attendance at their advisory meetings a faculty active in the IMSD program and a program representative to ensure adequate academic advisory support is provided to each student. IMSD trainees also subscribe to training modules designed to address gaps in background academic preparation that could threaten academic success. Adherence to these practices is optional for non-IMSD students, and thus, potential needs and gaps in their background preparation are not always addressed. Additionally, *en route* to completing their Ph.D. degrees, IMSD trainees must also submit advisor-approved written research summaries. This helps to ensure close and regular communication between trainee and advisee and timely work progress and helps to maintain scholarly excellence and rigor throughout training. The latter is also not a uniform requirement of other graduate students, and this may reflect the difference in their progress. IMSD trainees also take advantage of opportunities to build community by holding regular student-led community meetings and events that extend their networks of peers, near peers, and mentors. These include activities through a variety of university and IMSD sponsored groups such as Brown’s SACNAS chapter and at national conferences. We also believe that faculty attention and engagement, a requirement of the compact for IMSD program participation, also contributes to the lower student attrition rate. Engagement by faculty is an important component of the co-curricular scaffold we have built around our students. We also note a lower rate of attrition among non-IMSD URM students relative to the larger student population. We believe that this is due to a halo effect that comes from the higher group affinity between non-IMSD URM and IMSD trainees. Non-IMSD URMs also appear to subscribe to IMSD programming at a higher rate than other students which may help to reduce attrition.

The average number of publications produced by IMSD trainees equals that produced by the matched non-IMSD cohort (Figure 5). Both groups also produce equivalent numbers of first-author manuscripts with only a small statistically insignificant variance between the two. This variance exists because more matched cohort students coauthor the same manuscripts, lessening their opportunities at first-author publications. Interestingly, and in contrast to the matched cohort, no two IMSD trainee coauthor any of the publications reported for this group in this study. Finally, we note that 37 of the 365 publications attributable to the matched cohort list 2 or more matched cohort trainees as coauthors. When these duplicate or triplicate publications are accounted for, the average publication per matched cohort is calculated to be 2.7.

Twenty-eight IMSD trainees have completed Ph.D. training since 2008. Prior to completion, two trainees transferred from Brown for nonacademic reasons, completing their master’s at Brown and subsequently completing their Ph.D. degrees at other institutions. Former program trainees include individuals currently holding ranks of Associate and Assistant Professors at Carnegie-classified R1 institutions, master’s institutions, and 4-year teaching-intensive baccalaureate institutions. Past trainees who elected to pursue nonacademic careers continue to be “field-active” scholars and practitioners, using their degrees in the life, biomedical, and public health sciences.

The successful post-training placement of IMSD program alums suggests that their training has equipped them with the skills needed to be successful STEM field-active scholars. The observation that some former trainees pursue careers outside of the academy is not inconsistent with national trends among advanced STEM degree recipients (Sauermann and Roach, 2012). Technological innovations of the biotech and genome era have led to the rapid expansion of nonacademic career pathways in applied sciences fields. This career choice also reflects the ability of the job market to respond to the growing trend and desire of graduates to engage in public scholarship that is of immediate benefit to society. The relationship of post-training placement to graduate training reflects a strong alignment of graduate program curricula to prepare for these career opportunities. This alignment alone however is not sufficient for success. In addition to training excellence, excellent placement requires that trainees have access to and develop an awareness of career opportunities that come in part from attendance at local, regional, and national conferences and professional society meetings. IMSD and the university has helped trainees build the extended networks needed to support their post-training placement by supporting their attendance at many of these meetings.

Annually, we rely on climate survey responses to institute changes in training practices to benefit students. The almost uniformly positive responses by IMSD trainees to the climate in their programs are outcomes ascribed to the level of support provided by IMSD and faculty which we believe can be broadly applied to other STEM and non-STEM programs across the university. Constant changes in student and faculty populations makeup signify that improvements in institutional climate do not take place in a closed system with a fixed population. As a result, climate improvements are not always recognized in survey responses. Despite this, it is evident that the highest level of satisfaction with climate is seen among IMSD trainees.

While the results presented point to IMSD-associated improvements in diversity over the past decade, success has thus far been limited to the BioMed division. The expansion of IMSD in 2016, which aligned its work with broader university diversity practices, was therefore implemented to strengthen diversity across the campus. This expansion was enabled by Brown’s strategic plan for diversity, the DIAP, as well as support from the Division of BioMed and leadership changes in the Graduate School. Implementation and change placed greater emphasis on academic excellence that draws on and benefits from increased compositional diversity among STEM graduate students. Centered on Brown’s unifying principles of diversity and inclusion, new BioMed and Graduate School programming and resources were created and existing practices strengthened. This work benefited from one of IMSD’s Co-PI simultaneously serving as dean of the Graduate School and as a member of Brown’s inaugural DIAP Reports Review Committee. Within the framework created by Brown’s DIAP and programming across the Division of BioMed and the Graduate School, the application of IMSD practices has helped to increase diversity in both STEM and non-STEM graduate programs.

While it is too early to assess the full benefits of IMSD expansion, a signal of its impact is evident in the increased numbers of URM applicants to the Graduate School and STEM programs outside of BioMed. This observation is consistent with the belief that the combination of the work of the IMSD program work and institutional commitment to diversity would make Brown a more desirable university for URM graduate training. This is further confirmed by the observed continued increases in URM applicants, accompanied by record numbers of admitted and matriculating URM students to both master’s and Ph.D. programs. Early programming responsible for this outcome includes both Preview Day and Super Monday, both of which have helped to build student communities before matriculation.

We anticipate a more modest increase in BioMed diversity in the future which is consistent with a natural “ceiling effect.” We do, however, foresee that the level of diversity across the university, outside of BioMed, will increase more rapidly and may eventually approach the level achieved in the Division of BioMed.

**Fig. 6:**
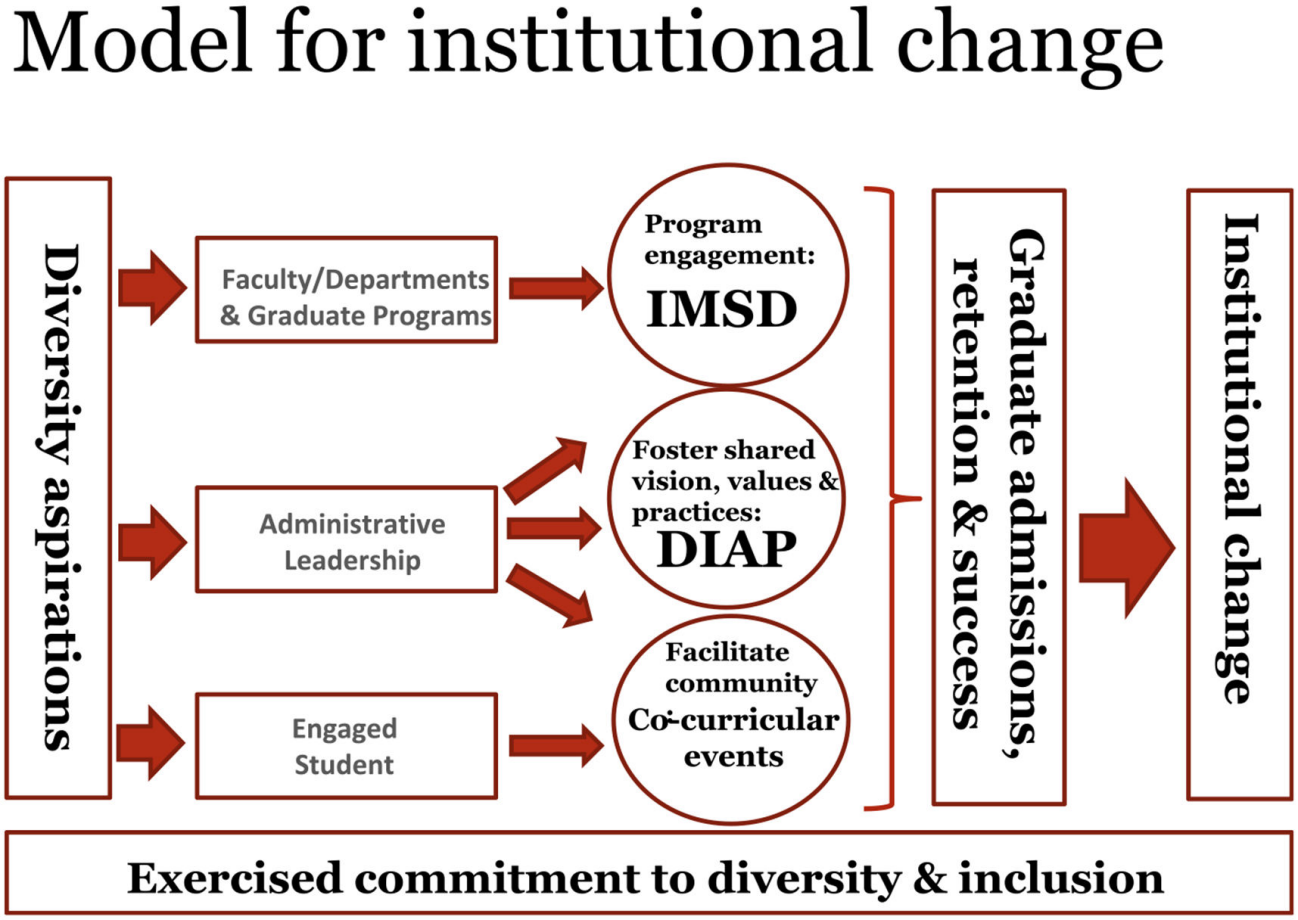
Working model illustrating interventions leading to increased underrepresented student matriculation and success in graduate training. Model highlights defining diversity aspirations as guides to achieving institutional change (vertical boxes). Listed in horizontal boxes are specific internal institutional stakeholders and partners contributing to programs and programming (circles: IMSD, DIAP, and co-curricular events)

Graduate student success is strongly influenced by early and adequate preparation and acclimation to the environment. It is maximized by providing students with adequate time and support resources, especially if these have been limited in the past. Matriculating graduate students also often lack the experiential knowledge of the graduate culture and early supportive framework and are thus not always equipped with the skill sets to maximize their success. This is often reflected in attrition from graduate studies, longer time to the degree, and other poor outcomes—much of these disproportionately affecting UR students. This study shows the benefits of establishing programming designed to maximize student training success and post-training placement and to build a sense of belonging. The results show that well-designed and sustained programming can maximize success. Figure 6 presents a model that summarizes how institutional change is being achieved at Brown. Success over the last decade has come about as a result of careful coordination and collaboration across the Brown community by engagement of all stakeholders. The implementation of many of IMSD practices requires close collaboration with faculty in their roles as trainers, mentors, and academic program leaders. Other programs have employed similar practices with great success. One of these, the Meyerhoff Scholar Program (MYS) at the University of Maryland, Baltimore County (https://meyerhoff.umbc.edu/), supports the training of underrepresented undergraduate students. This work has resulted in student academic success as measured in part by their progression to advanced graduate and professional degree training (Summers and Hrabowski 3rd., 2006). MYS has demonstrated the sustainability and transferability of its practices by replicating its success at other institutions (Sto Domingo et al., 2019).

A 10-year analysis of performance and outcomes measures of trainee success in the University of Arkansas for Medical Sciences (UAMS) graduate-level IMSD program was recently completed (Williams et al., 2021). Among the many measures were analyses including examination of correlations between quantitative GRE scores and first-author publications, first-author publications and first-semester Graduate School GPA, and time to degree with standardized test scores. While our analyses did not examine these correlations, we did examine some of the same performance and outcomes measures. Overall, UAMS reported that, despite the lower GPA and GRE credentials, trainees from historically black colleges and universities (HBCUs) performed as well as trainees from predominantly white institutions (PWIs), attributing student success to a number of their IMSD interventions. They also report that over the life of their students’ graduate careers, there was no statistical difference in the performance of students from HBCUs and PWIs. These findings are consistent with our observations for Brown IMSD trainees and the matched cohort, which we also attribute to preparatory training offered by our program.

The framework for success, including local and institutional climate change, is shaped by administrative leadership. Equally important is the role of students as accountable partners in their training. Many of the practices and programming described here are not unique to Brown IMSD nor are they restricted to any one group of students. We hope that many of the practices described here may be applied elsewhere to achieve similar levels of graduate diversity and student success.

## Supplementary Material

Suppl Figure

## Data Availability

The datasets generated during and/or analyzed during the current study are available from the corresponding author on reasonable request.
