# Supplementary material for: Improved and Sustained Graduate Programs Diversity Outcomes: a 10-year Analysis and Summary of the Brown University IMSD Program
Source: J STEM Educ Res. Author manuscript; Available in PMC 2021 Dec 28. (PMC8713804; doi:10.1007/s41979-021-00057-z)
Supplement: Suppl Figure [file NIHMS1763072-supplement-Suppl_Figure.pptx]

## Slide 1
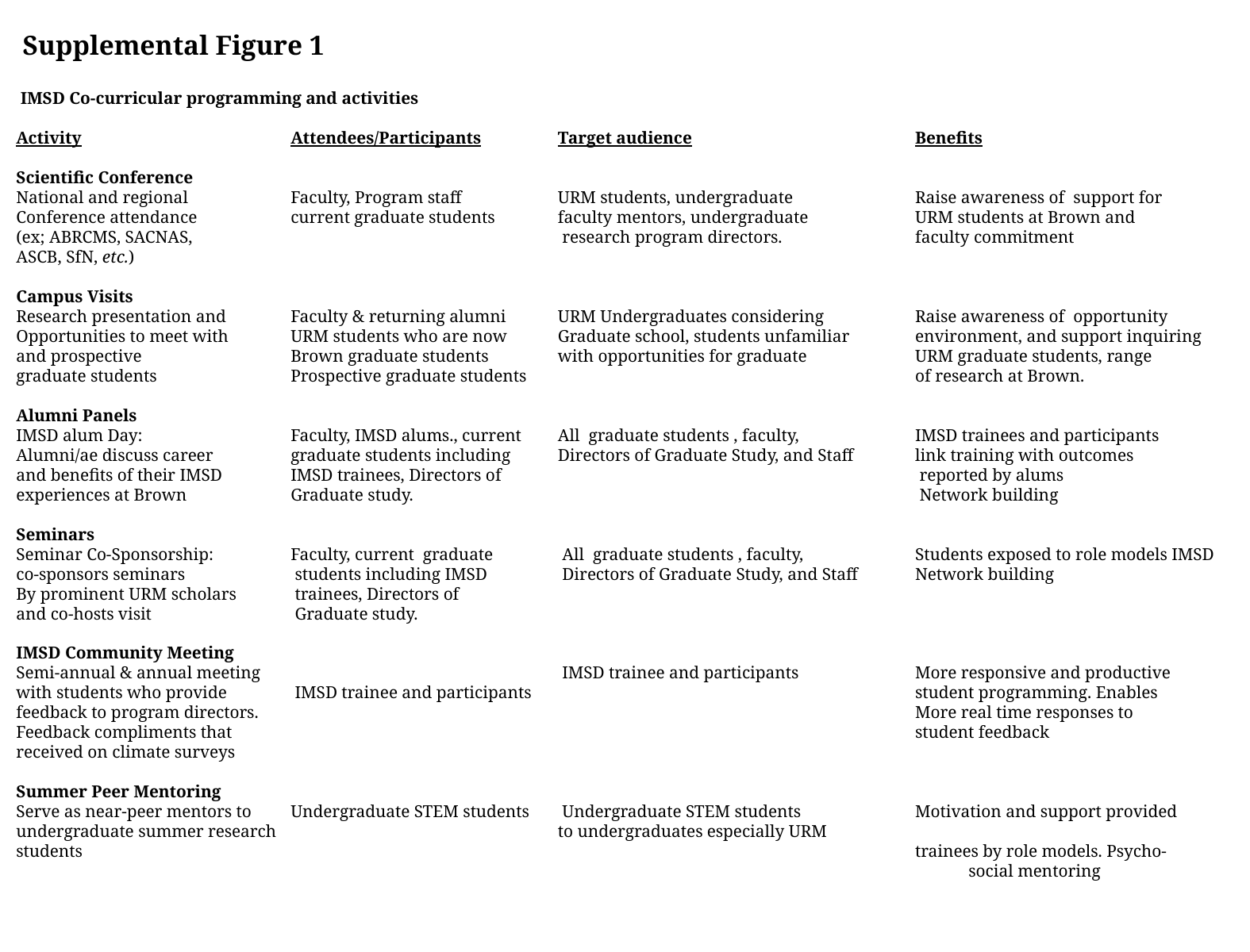

Supplemental Figure 1
 IMSD Co-curricular programming and activities
Activity	Attendees/Participants	Target audience	Benefits
Scientific Conference
National and regional	Faculty, Program staff	URM students, undergraduate 	Raise awareness of support for
Conference attendance 	current graduate students 	faculty mentors, undergraduate 	URM students at Brown and
(ex; ABRCMS, SACNAS, 		 research program directors.	faculty commitment
ASCB, SfN, etc.)
Campus Visits
Research presentation and	Faculty & returning alumni	URM Undergraduates considering	Raise awareness of opportunity
Opportunities to meet with	URM students who are now	Graduate school, students unfamiliar 	environment, and support inquiring and prospective	Brown graduate students	with opportunities for graduate 	URM graduate students, range
graduate students	Prospective graduate students		of research at Brown.
Alumni Panels
IMSD alum Day:	Faculty, IMSD alums., current 	All graduate students , faculty, 	IMSD trainees and participants Alumni/ae discuss career	graduate students including	Directors of Graduate Study, and Staff 	link training with outcomes
and benefits of their IMSD	IMSD trainees, Directors of	 	 reported by alums
experiences at Brown	Graduate study. 		 Network building
Seminars
Seminar Co-Sponsorship:	Faculty, current graduate	 All graduate students , faculty, 	Students exposed to role models IMSD co-sponsors seminars	 students including IMSD	 Directors of Graduate Study, and Staff 	Network building
By prominent URM scholars	 trainees, Directors of
and co-hosts visit	 Graduate study.
IMSD Community Meeting
Semi-annual & annual meeting	 	 IMSD trainee and participants	More responsive and productive
with students who provide	 IMSD trainee and participants 		student programming. Enables
feedback to program directors.	 		More real time responses to
Feedback compliments that			student feedback
received on climate surveys
Summer Peer Mentoring
Serve as near-peer mentors to	Undergraduate STEM students 	 Undergraduate STEM students 	Motivation and support provided
undergraduate summer research 		to undergraduates especially URM
students 			trainees by role models. Psycho-
				social mentoring

## Slide 2
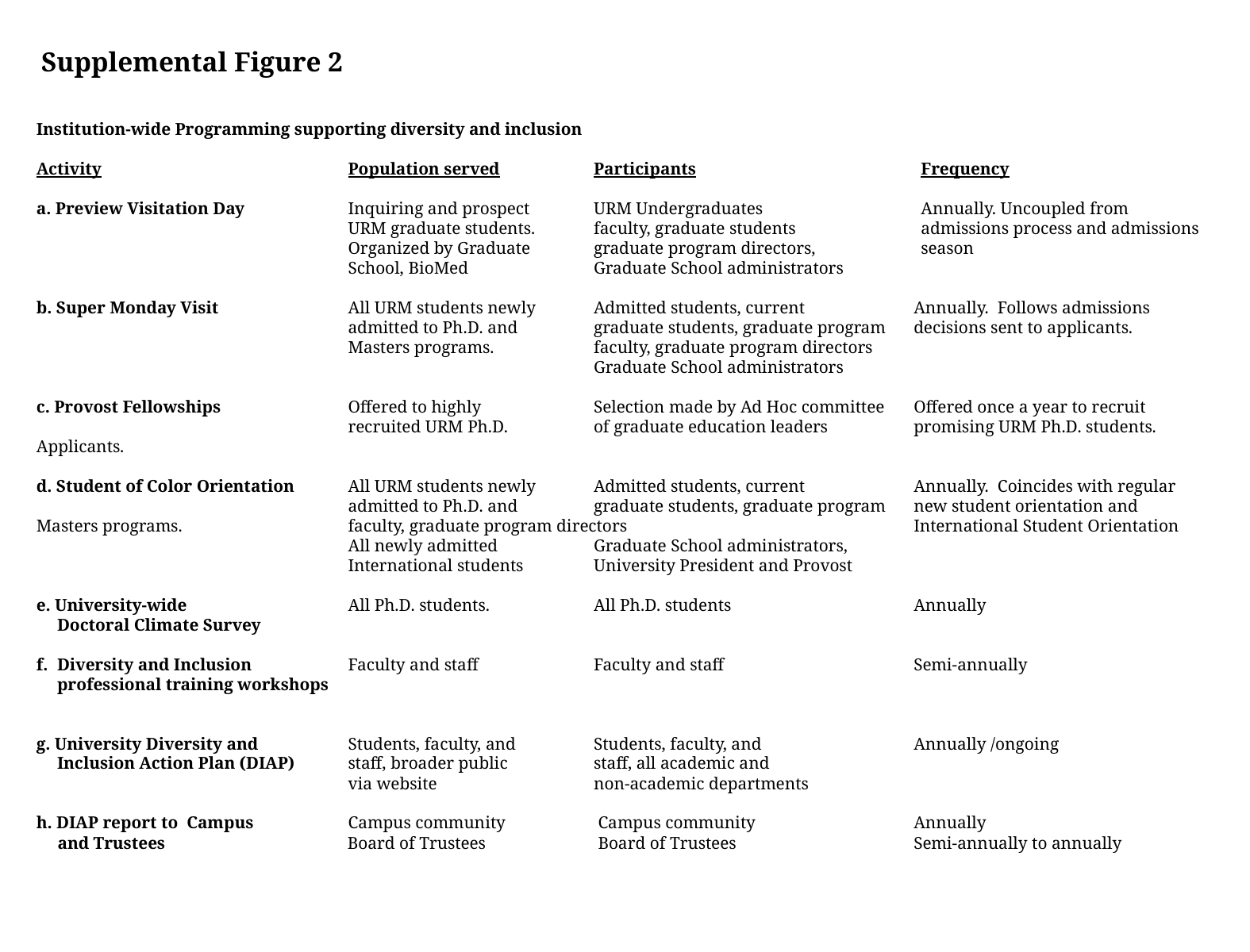

Supplemental Figure 2
Institution-wide Programming supporting diversity and inclusion
Activity	Population served	Participants	Frequency
a. Preview Visitation Day	Inquiring and prospect	URM Undergraduates	Annually. Uncoupled from
	URM graduate students.	faculty, graduate students	admissions process and admissions
	Organized by Graduate 	graduate program directors,	season
	School, BioMed	Graduate School administrators
b. Super Monday Visit	All URM students newly 	Admitted students, current 	Annually. Follows admissions
	admitted to Ph.D. and 	graduate students, graduate program 	decisions sent to applicants. 		Masters programs. 	faculty, graduate program directors
		Graduate School administrators
c. Provost Fellowships	Offered to highly 	Selection made by Ad Hoc committee	Offered once a year to recruit
	recruited URM Ph.D. 	of graduate education leaders 	promising URM Ph.D. students. 	Applicants.
d. Student of Color Orientation	All URM students newly 	Admitted students, current 	Annually. Coincides with regular
	admitted to Ph.D. and 	graduate students, graduate program 	new student orientation and 	Masters programs. 	faculty, graduate program directors 	International Student Orientation
	All newly admitted 	Graduate School administrators,
	International students	University President and Provost
e. University-wide 	All Ph.D. students. 	All Ph.D. students	Annually
	Doctoral Climate Survey
f.	Diversity and Inclusion	Faculty and staff 	Faculty and staff 	Semi-annually
	professional training workshops
g. University Diversity and 	Students, faculty, and 	Students, faculty, and 	Annually /ongoing
	Inclusion Action Plan (DIAP)	staff, broader public 	staff, all academic and
	via website	non-academic departments
h. DIAP report to Campus 	Campus community	 Campus community 	Annually
	and Trustees 	Board of Trustees	 Board of Trustees 	Semi-annually to annually
